# Neuropeptide Y in the Adult and Fetal Human Pineal Gland

**DOI:** 10.1155/2014/868567

**Published:** 2014-03-17

**Authors:** Morten Møller, Pansiri Phansuwan-Pujito, Corin Badiu

**Affiliations:** ^1^Department of Neuroscience and Pharmacology, Laboratory of Neuropsychiatry, University of Copenhagen, 2100 Copenhagen, Denmark; ^2^Department of Anatomy, Faculty of Medicine, Srinakharinwirot University, Bangkok 10110, Thailand; ^3^National Institute of Endocrinology, “C. Davila” University of Medicine and Pharmacy, 050474 Bucharest, Romania

## Abstract

Neuropeptide Y was isolated from the porcine brain in 1982 and shown to be colocalized with noradrenaline in sympathetic nerve terminals. The peptide has been demonstrated to be present in sympathetic nerve fibers innervating the pineal gland in many mammalian species. In this investigation, we show by use of immunohistochemistry that neuropeptide Y is present in nerve fibers of the adult human pineal gland. The fibers are classical neuropeptidergic fibers endowed with large *boutons en passage* and primarily located in a perifollicular position with some fibers entering the pineal parenchyma inside the follicle. The distance from the immunoreactive terminals to the pinealocytes indicates a modulatory function of neuropeptide Y for pineal physiology. Some of the immunoreactive fibers might originate from neurons located in the brain and be a part of the central innervation of the pineal gland. In a series of human fetuses, neuropeptide Y-containing nerve fibers was present and could be detected as early as in the pineal of four- to five-month-old fetuses. This early innervation of the human pineal is different from most rodents, where the innervation starts postnatally.

## 1. Introduction

The pineal gland of mammals is an endocrine gland secreting the hormone melatonin with a circadian rhythm exhibiting a zenith during the night time [1, 2]. The synthesis of melatonin is regulated by a dense network of sympathetic nerve fibers originating from perikarya located in the autonomic superior cervical ganglia [3, 4]. Most of these sympathetic nerve fibers do also costore neuropeptide Y (NPY).

NPY was originally isolated from the porcine hypothalamus [5] and later found to be colocalized with noradrenaline in most sympathetic fibers [6] including the nerve fibers innervating the rat pineal gland [6–10].

The sequence of the NPY gene encodes a pre-pro NPY, a precursor peptide of 97 amino acids, which is cleaved into the pro-NPY, a 69-aa peptide [11]. The pro-NPY molecule is posttranslationally processed by a single cleavage to neuropeptide Y (NPY) and a C-terminal peptide of NPY (CPON). NPY is Cterminally amidated, and the amidation is essential for binding of NPY to its corresponding receptors [12].

A few of the NPYergic nerve fibers innervating especially the rostral part of the rat pineal gland do not originate from perikarya in the superior cervical ganglia [8, 9, 12, 13]. Thus, some NPY-containing nerve fibers remain in the gland after bilateral superior cervical ganglionectomy [12] and these fibers might originate from neurons located in the brain itself (pineal central innervations). Ultrastructural analysis using immunocytochemistry has shown NPY to be confined in the large dense core granules in the sympathetic nerve terminals [12].

The NPYergic innervation of the mammalian pineal has later been confirmed in numerous mammalian species, for example, cotton rat [14], Golden hamster [15], European hamster [16], guinea pig [17], gerbil [13], mink [18], chinchilla [19], tree shrew [20], brown bat [21], sheep [22, 23], cat [24], pig [25, 26], cow [27, 28], and monkey [29].

Release of noradrenaline in the pineal gland starts a biochemical cascade, which includes binding of the transmitter to beta-adrenergic receptors and initiation of the cAMP second messenger system, which has been thoroughly described [30]. However, what happens after release of NPY in the pineal gland is still a matter of discussion.

Melatonin is also synthesized in and secreted from the human pineal gland [31] and removal of the sympathetic input to the gland inhibits the elevated night time excretion of the hormone [32]. There has been a single report on the presence of a few NPY-immunoreactive fibers in the human pineal gland [33]. We have in this investigation performed a detailed study of the presence of the NPYergic innervations in adult human pineal obtained at autopsy and included a series of human fetal pineal gland obtained by caesarian sections. We show the presence of dense NPYergic innervations in adult pineal gland. An NPYergic innervation is also present in human fetal pineals where the first immunoreactive nerve fibers were observed in the 4th fetal month.

## 2. Materials and Methods

### 2.1. Antisera and Peptides

The primary Rabbit antisera against NPY used in the present investigation (numbers 8182 and 337) have been characterized previously [34, 35]. Antisera against the C-terminal flanking peptide of NPY (CPON) were purchased from Cambridge Research Biochemical (Cheshire, UK).

Biotinylated swine antirabbit IgG was obtained from Dako, Glostrup, Copenhagen (number E353). The ABC-streptavidin-horseradish peroxidase complex was obtained from Vector, Burlingame, CA, USA. (Vectastain Elite ABC kit, number PK-6100)

### 2.2. Adult Human Pineal Gland

A series of human pineal glands of both sexes were obtained from hospital autopsies. The age varied from 6 to 82 years. The postmortem time before fixation varied from 12 to 36 hours.

The brains were removed and the epithalamic area was dissected from the brain and immersed in cold (4°C) 4% paraformaldehyde in 0.1 M phosphate buffer (pH 7.4) for 5 days. The tissue blocks were then infiltrated with a solution of 30% sucrose in phosphate buffered saline (PBS) for 3 days and 18 *μ*m or 40 *μ*m thick serial cryostat sections were cut into sagittal and coronal planes. The 40 *μ*m thick sections were transferred to PBS at 4°C. The 18 *μ*m thick sections were placed on gelatinized glass slides.

### 2.3. Human Fetal Brains

The material examined was obtained from legal abortions and consisted of pineal glands from human fetuses of both sexes. The gestational ages were from 2.5 months (9 weeks) to 6 months (24 weeks). The postmortem interval preceding fixations varied, but in most cases the brains were immersed in cold (4°C) 4% paraformaldehyde in 0.1 M phosphate buffer (pH 7.4) for less than 30 minutes after removal from the uterus. Two extensive sagittal cuts were made in each hemisphere to ensure good penetration of the fixative into the brain ventricular system. The brains were cryoprotected in 30% sucrose as described above, frozen in crushed CO_2_, and cut into sagittal or coronal, 18 *μ*m thick, cryostat sections.

### 2.4. Immunohistochemistry

The sections were processed for immunohistochemistry by the use of the streptavidin enzyme histochemical technique. The 40 *μ*m thick sections were reacted as free floating sections and the 18 *μ*m thick sections were reacted on the glass slides in a humid chamber. The sections were rinsed twice for 5 min in PBS and pretreated in 1% H_2_O_2_ in PBS for 10 min. They were then incubated for 20 min in a 4% swine serum solution in PBS containing 0.3% Triton X-100 and 1% bovine serum albumin. This was followed by incubation in the primary antiserum for 24 h at 4°C. The dilutions were as follows: NPY (antisera numbers 337 and 8182), 1 : 1,000; CPON, 1 : 2,000. The sections were then washed in PBS to which 0.25% bovine serum albumin and 0.1% Triton X-100 were added (PBS-BT) for 3 × 10 min followed by incubation with the biotinylated swine anti-rabbit IgG, diluted 1 : 400 in PBS-BT, for 60 min at room temperature. They were next washed for 3 × 10 min in KPBS-BT and finally incubated for 60 min at room temperature in an ABC-streptavidin-horseradish peroxidase complex 1 : 500 in PBS-BT. After washing in KPBS-BT for 10 min, in KPBS alone for 10 min, and in 50 mM Tris/HCl buffer (pH 7.6) for 10 min, the sections were reacted for peroxidase activity by incubation with a solution of 1.25 mg/L diaminobenzidine (DAB) in 0.05 M Tris/HCl-buffer (pH 7.6) and 0.003% H_2_O_2_ for 20 min. After washing for 2 × 5 min in distilled water, the sections were mounted on gelatinized glass slides, dried, dehydrated in a series of ethanols, and embedded in Depex. Some adjacent sections were counterstained in thionine after the DAB reaction.

Absorption controls were done by substituting the primary antisera with antisera preabsorbed for 48 hr with the specific antigen (100 *μ*g/mL diluted antiserum) at 4°C.

## 3. Results

### 3.1. Adult Human Pineal Glands

**Figure 1:**
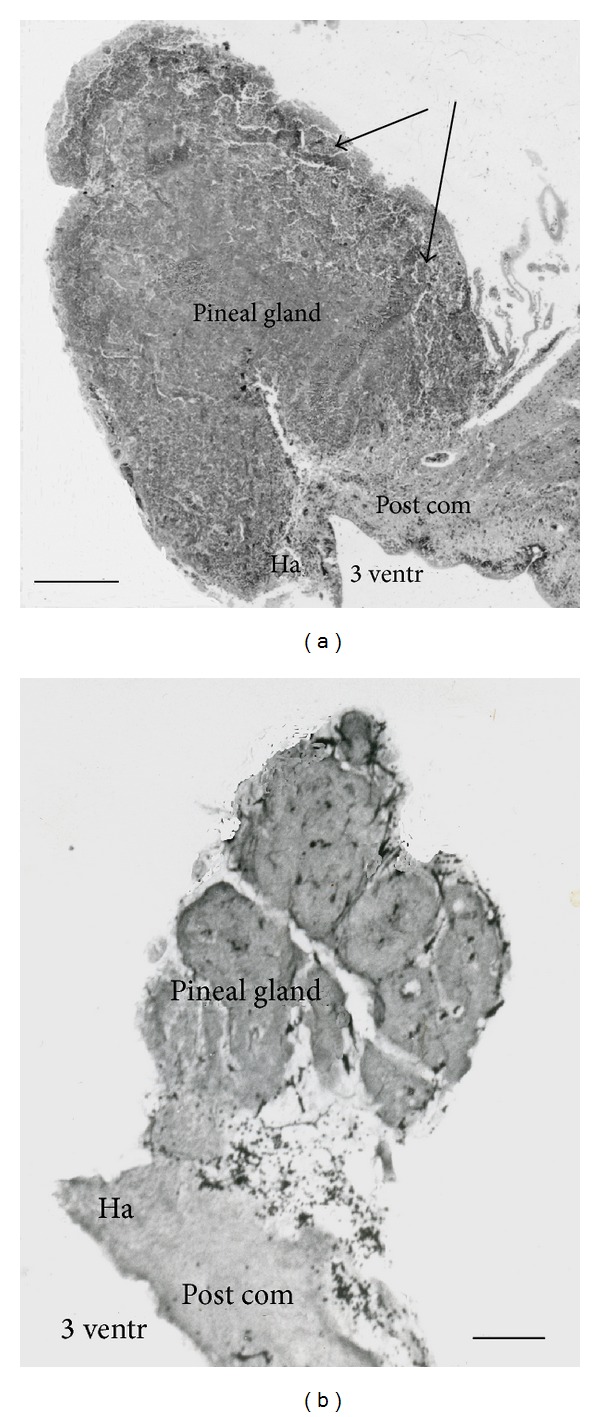
(a) Sagittal section of a human pineal gland of a young adult obtained at autopsy. The pineal recess is seen below the gland and some pial tissue is located caudal to the gland. Note the follicular appearance of the pineal parenchyma (arrows). Bar = 1 mm. (b) Sagittal section of a 7-month-old human fetus. Note the follicular appearance of the parenchyma. Bar = 250 *μ*m. Ha: habenula, Post com: posterior commissure, and 3 ventr: third ventricle.

The adult human pineal is located on the dorsal part of the brain stem at the mesodiencephalic border connected to the epithalamic area with a short broad stalk (Figure 1(a)). The gland varies in size and often develops calcifications in adult; it is about 12 mm height, 7 mm in width, and about 6 mm in anterior-posterior direction. The third ventricle makes an evagination; the pineal recess into the stalk and separates the rostral habenular area from the posterior commissure caudal to the gland.

The pineal parenchyma, consisting of pinealocytes, interstitial cells, phagocytes, and capillaries, is arranged into large folliculi separated by septae of connective tissue and blood vessels. 

**Figure 2:**
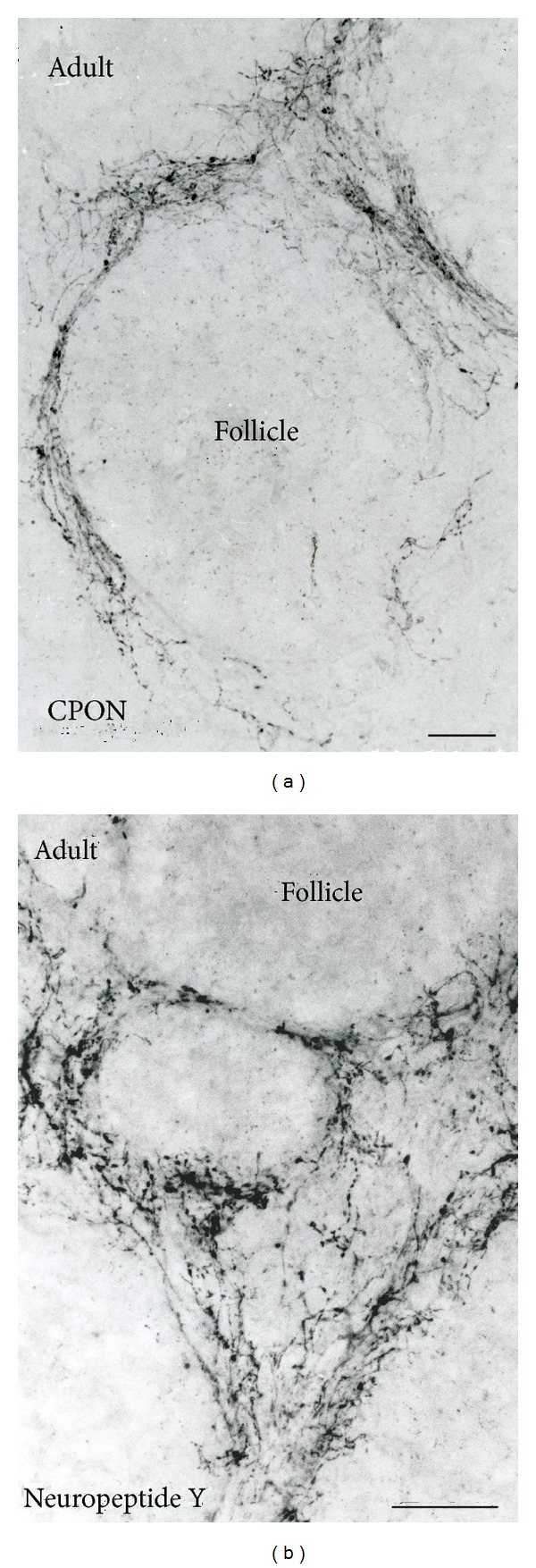
(a) and (b) Photomicrographs of parts of the adult human pineal gland obtained at autopsy, which have been immunoreacted for CPON (a) and NPY (b). Note in both pineals the perifollicular location of NPY-immunoreactive nerve fibres with large* boutons en passage*. Several of the nerve fibres penetrate into the follicle. Bars = 250 *μ*m ((a) and (b)).

Numerous NPY-immunoreactive nerve fibers endowed with large* boutons en passage *were present in a perifollicular position (Figures 2(a) and 2(b)). Some of the immunoreactive nerve fibers penetrated into the follicle itself, but a dense innervation of the follicle itself was not present.

Several immunoreactive nerve fibers were present in the pineal stalk and some of these immunoreactive nerve fibers entered the rostral part of the pineal gland.

### 3.2. Fetal Pineal Glands

**Figure 3:**
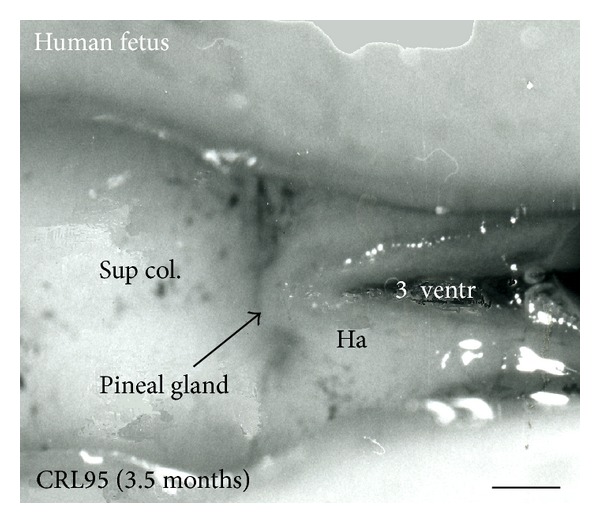
Superior (dorsal) view of the diencephalic-mesencephalic area of a 3.5-month-old human fetus. The third ventricle (3 ventr) without pial covering is seen to the right in the micrograph. The small pineal gland is a small protuberance (arrow) and merging via the broad stalk with the habenula (Ha). Sup col.: superior colliculus. Bar = 2 mm.

The human fetal pineal gland develops in the second month of gestation as an evagination of the ependyma covering the third ventricle at the diencephalic-mesencephalic border [36, 37]. The cell proliferation of the ependymal cells gives rise toto an anterior- and posterior "anlage" (lobe) which later are merging. In the third gestational month, the pineal is macroscopically visible (Figure 3). The follicular appearance of the pineal parenchyma is clearly visible (Figure 1(b)).

**Figure 4:**
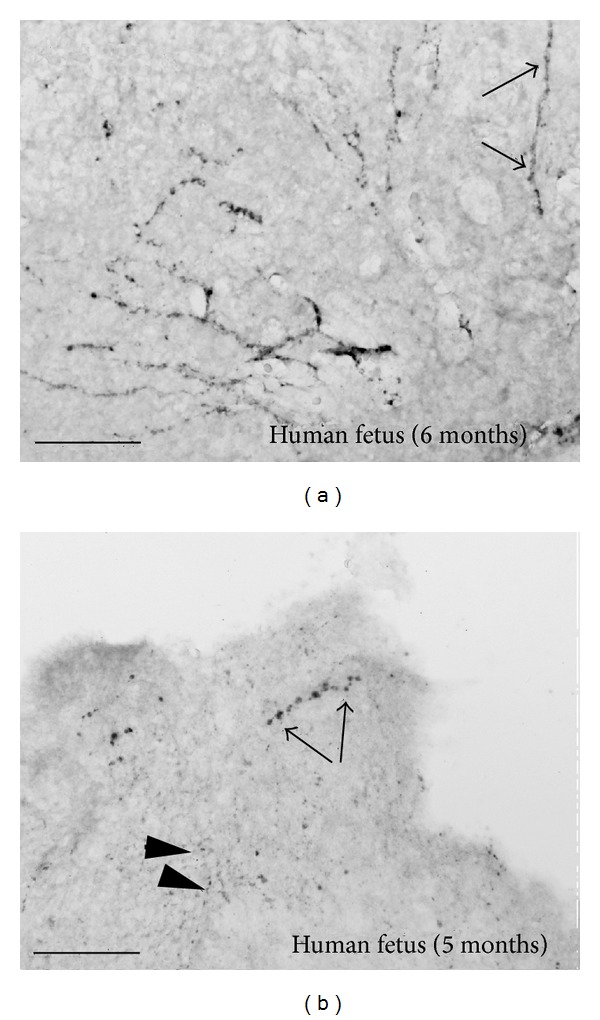
Photomicrographs of parts of human fetal pineal glands reacted immunohistochemically for neuropeptide Y. (a) Pineal gland from 6-month-old fetus. Arrows mark a long NPY-immunoreactive nerve fiber with large* boutons en passage*. (b) Pineal gland from 5-month-old fetus. Arrows mark an immunoreactive nerve fiber with large* boutons en passage*. Arrow heads point toward smaller immunoreactive boutons. Bar = 200 *μ*m.

The NPYergic innervation of the fetal pineal with gestational ages of 6 to 7 months was quite dense (Figure 4(a)). The number of NPYergic immunoreactive nerve fibers declined in the 5-month-old fetuses (Figure 4(b)), and in the 4th gestational month only few and thin immunoreactive fibers were observed. In our series, we did not stain immunoreactive nerve fibers in fetuses younger than 4th gestational month.

There was a clear relationship between the postmortem time of the tissue before fixation and the immunoreactivity of the nerve fibers in the tissue sections. The morphological best fibers were obtained in the pineals which were fixed with the shortest time delay.

## 4. Discussion

We show in this paper dense NPYergic innervations of both the adult and fetal human pineal glands with classical neuropeptidergic nerve fibers endowed with large* boutons en passage*. The prominent perifollicular distribution of the nerve fibers in the human is different from the distribution seen in several other rodent and nonrodent species, where many of the NPYergic nerve fibers penetrate into the pineal parenchyma between the pinealocytes. Due to the delay caused by the diffusion distance from the perifollicular terminals to the pinealocytes, this might indicate a modulatory function for NPY in human pineal biochemistry. The majority of the NPYergic nerve fibers are probably originating from sympathetic nerve fibers in the superior cervical ganglion. However, the evidence of a sympathetic innervation of the human pineal gland is indirect because a superior cervical ganglionectomy in humans has never been performed. However, in tetraplegic patients, with total transverse lesions of the cervical spinal cord, the night time elevation of plasma melatonin is abolished [38–41]. This indicates that intact nerve fibers in the cervical parts of the spinal cord are a prerequisite for the presence of a circadian rhythm of melatonin secretion. Further, transection of the sympathetic trunk at the level of the second thoracic ganglion in patients to prevent hyperhidrosis abolishes the night time elevation of melatonin secretion in the majority of these patients [32].

The current study also showed many NPYergic nerve fibers in pineal stalk of both the adult and fetal pineals and might indicate the presence of NPYergic nerve fibers innervating of the human pineal from the brain via the pineal stalk. Such a central pineal innervation in mammals has been a matter of controversy. Early studies in humans described the presence of silver stained nerve fibers penetrating into the pineal gland via the stalk from the habenular and posterior commissures [42]. However, in later studies of the rat it was suggested that these fibers looped in the rostral part of the pineal and returned to the brain without making functional contacts [43]. Contrarily, lesions of the habenular area in the rat resulted in anterograde degenerating nerve fibers in the pineal gland [44]. Later, retro- and anterograde* in vivo* tracings of fibers innervating the pineal gland showed the origin of these central fibers to be located in the paraventricular nucleus [45] as well as in neurons located in the lateral geniculate body of the thalamus [46, 47]. In rodents, the intergeniculate leaflet of the lateral geniculate body contains NPYergic neurons, which projects to the SCN and is responsible for a nonphotic regulation of the endogenous clock [48]. It is possible that some NPYergic neurons in the intergeniculate leaflet also project to the pineal gland.

A parasympathetic innervation of the pineal gland is also present [49, 50]. NPY also been found to be colocalized with acetylcholine in parasympathetic ganglia known to project to the pineal gland [51]. Therefore, the parasympathetic system might contribute to the pineal NPYergic innervation.

The physiological function of NPY in the pineal gland is not clear. There is no direct stimulatory effect of NPY on the secretion of melatonin in cultures, but NPY has an indirect effect by inhibiting the stimulatory effect of noradrenaline on melatonin release [52, 53]. The effect of NPY on the noradrenergic transmission is probably transmitted via inhibitory G-proteins in the membrane reducing the activity of adenylate cyclase in the target cells [54, 55]. The cells possessing receptors for NPY in the pineal gland have not been determined, but specific binding sites have been demonstrated in suspensions of cultured pineal cells [52], indicating that postsynaptic receptors are present in the pineal gland. Further evidence has shown that the NPY binding site is of the Y1 subtype [53], which is supported by reverse-transcriptase polymerase chain reaction studies, showing that only Y1 mRNA, and not any of the other subtypes (Y2, Y4, or Y5) was expressed [56].

The detection of weakly stained NPYergic fibers in four-month-old fetal pineal is in accord with studies on NPY in other regions of the brain, demonstrating that NPY can be detected around the 4th fetal month and that the number of NPYergic neurons increases with fetal age [57]. In a study of human fetal spinal cords NPY-immunoreactive neurons were found in the dorsal horn as early as 10 weeks of fetal age [58].

From the physiological point of view there are indications in the European hamster* (Cricetus cricetus)* that NPY might control annual rhythms. Thus, the number of NPYergic intrapineal nerve fibers exhibits an annual rhythm with a zenith in midwinter [16]. At midwinter 5-methoxytryptophol starts to exhibit a nycthemeral rhythm and the activity of hydroxyindole-O-methyltransferase (HIOMT), a key enzyme in the synthesis of melatonin, is significantly increased [59]. In the rat, NPY stimulates HIOMT activity [60]. If NPY also stimulates HIOMT in the European hamster, NPY might be directly involved in the annual regulation of the pineal gland in this species.

In summary, this paper shows the presence of dense innervations of the human pineal gland with classical neuropeptidergic NPY-immunoreactive nerve fibers. The NPYergic innervation of the human pineal gland starts in fetal life at about the 4th month of gestation.
